# Satisfaction with fertility- and sexuality-related information in young women with breast cancer—ELIPPSE40 cohort

**DOI:** 10.1186/s12885-015-1542-0

**Published:** 2015-08-05

**Authors:** Ali Ben Charif, Anne-Déborah Bouhnik, Dominique Rey, Magali Provansal, Blandine Courbiere, Bruno Spire, Julien Mancini

**Affiliations:** 1 UMR912 “Economics and Social Sciences Applied to Health & Analysis of Medical Information” (SESSTIM), 13006, Marseille, France; 2 UMR_S912, IRD, Aix Marseille Université, Marseille, France; 3 ORS PACA, Southeastern Health Regional Observatory, Marseille, France; 4 Institut Paoli-Calmettes, Marseille, France; 5 IMBE UMR7263, Aix Marseille Université, CNRS, IRD, Avignon Université, Marseille, France; 6 Department of Obstetrics, Gynecology and Reproductive Medicine, APHM, La Conception Hospital, Marseille, France; 7 BiosTIC, La Timone Hospital, APHM, Marseille, France; 8 UMR912, SESSTIM, “Cancers, Biomedicine & Society” group, Institut Paoli-Calmettes, 232 Bd Ste Marguerite, 13273 Marseille, France

**Keywords:** Breast cancer, Young women, Satisfaction, Fertility- and sexuality-related information, Prospective cohort, ELIPPSE40

## Abstract

**Background:**

Young breast cancer survivors are often dissatisfied with the information provided on fertility and sexuality. Our aim was to discuss possible contributing factors and to propose strategies to increase patient satisfaction with such information.

**Methods:**

Using the French National Health Insurance System database, we constituted the ELIPPSE40 regional cohort of 623 women, aged 18–40, diagnosed with breast cancer between 2005 and 2011. As of January 2014, 319 women had taken part in the 10-, 16-, 28 and 48-month telephone interviews. Satisfaction with the information provided about the potential impact of cancer and its treatment on fertility and sexuality was assessed at 48 months after diagnosis on 5-point Likert scales.

**Results:**

Four years after diagnosis, only 53.0 and 42.6 % of women were satisfied with fertility- and sexuality-related information, respectively, without any significant change over the 2009–2014 period (*P* = 0.585 and *P* = 0.676 respectively). The two issues were moderately correlated (*ρ* = 0.60; *P* <0.001). General satisfaction with medical follow-up was the only common correlate. Irrespective of sociodemographic and medical characteristics, satisfaction with fertility-related information was greater among women with a family history of breast/ovarian cancer who had the opportunity to ask questions at the time of cancer disclosure. Satisfaction with sexuality-related information increased with the spontaneous provision of information by physicians at cancer disclosure.

**Conclusions:**

Promoting both patients’ question asking behavior and more systematic information could improve communication between caregivers and young breast cancer survivors and address distinct unmet needs regarding fertility- and sexuality- related information.

**Electronic supplementary material:**

The online version of this article (doi:10.1186/s12885-015-1542-0) contains supplementary material, which is available to authorized users.

## Background

Thanks to early detection and treatment improvement, the mortality rate from Breast Cancer (BC) has declined over the past 20 years [1, 2]. Approximately 91 % of women with BC now survive for more than 5 years [2], although the prognosis appears to be worse in young women [3, 4]. Despite improved survival rates, the literature describes a wide range of difficulties in patients’ day-to-day lives arising from BC and associated treatment [5–8]. Young BC survivors face a multitude of challenges, including issues related to fertility and sexuality, which need to be addressed.

Among the drugs used in adjuvant therapy for BC, cyclophosphamide (an alkylating agent) is one of the most toxic for ovarian function [6, 9, 10]. Common effects of chemotherapy on ovarian function include temporary or permanent amenorrhea due to premature ovarian failure. Rates of amenorrhea can reach 80 % in women under 40 years of age with poor prognosis tumors who are prescribed more aggressive regimens, such as six cycles of fluorouracil plus epirubicin, doxorubicin or methotrexate and cyclophosphamide [6]. After 4–6 cycles of cyclophosphamide containing polychemotherapy, the ovaries of these young women age by approximately 10 years [10]. In addition, the duration of initial adjuvant hormone treatment (recommended for 5 years) prevents women from considering pregnancy, as fertility is likely to be reduced due to age-related decline. Accordingly, even though young women constitute a minority of BC patients (each year in France, approximately 10 % of newly-diagnosed women are under 40, [11]), they have specific concerns and issues, including queries regarding fertility.

Although sexual problems decrease over time and most women regain their previous sexual activity after treatment [12, 13], women of all ages report more sexuality-related difficulties (including decreased interest and sexual desire, anorgasmia, dyspareunia and lower levels of sexual satisfaction) when compared with their retrospective pre-diagnosis reports [14–18]. They are also more concerned about their sexuality than the general female population [13, 19, 20]. Sexual dysfunction is especially prominent among young women who are more vulnerable to changes in ovarian functioning resulting from chemotherapy and to concerns about body image after mastectomy [12, 18, 21, 22]. Sexual dysfunction is related to psychosocial outcomes, including depression, anxiety, and lower quality of life [21–23].

Unfortunately, many young women are not fully aware or well-informed about the potential adverse effects of BC treatment on fertility and sexual life [24, 25]. Today, the possibility of maintaining fertility and re-establishing an active sex life after cancer means that healthcare providers must provide adequate information about the effects of the disease and its treatments [24–26]. Some studies have shown that a lack of information regarding these issues can lead to increased patient uncertainty, anxiety, depression, distress, anger and confusion [27–31]. Conversely, good communication between patients and healthcare providers offers many advantages such as a greater satisfaction with healthcare providers, reduced distress and improved quality of life [24, 30–32]. One study found that the transmission of clear and complete information (for illness, medical examinations, treatment, cure, diagnosis, prognosis and side effects) improved the women’s quality of life up to 4 years after diagnosis [33]. Similarly, a positive association was found between satisfaction with information obtained and health-related quality of life dimensions: physical, functional, psychological and social [34]. Cancer survivors who were satisfied with the information provided by the physicians at diagnosis and during treatment had better mental health and vitality than those who were not satisfied with the information [35].

In France, the 1st and 2nd national cancer plans [36] have helped to standardize cancer diagnosis disclosure by healthcare providers and to improve patient information. However, unlike other industrialized nations [24, 25, 28, 37], French national guidelines regarding discussions and referral for fertility and sexual health in the oncology clinics are new, and have not been fully implemented [38, 39]. Their systematic adoption will not only ensure the provision of high quality information and the coordination of care, but will also prevent patients from being given conflicting advice about management of their symptoms. The 3rd French National Cancer Plan (in February 2014) provides specific recommendations about the management of infertility problems, but nothing about sexual issues.

In the context of the national effort to improve the quality and quantity of information provided after cancer diagnosis, the aims of the present study were to measure satisfaction with information provided on the topics of fertility and sexuality among young women with BC, to highlight factors contributing to this satisfaction and to propose possible strategies to increase it.

## Methods

### Study population

The Longitudinal Study on Psychosocial Impact of Breast Disease among women under 40 years (ELIPPSE40 cohort) is an ongoing regional observational study, first implemented in South Eastern France (PACA and Corsica regions) in September 2005, which documents the medium- and long-term consequences of BC and its treatments on women’s daily and social lives [40–43]. The study area covers a population of approximately five million inhabitants. All women with a diagnosis of biopsy-proven primary BC, aged 18–40, living in Southeastern France with a valid address, and included in the long-term disease registry of the French National Health Insurance Fund (NHIF) between 2005 and 2011 were eligible. Study inclusion ended in 2011. The NHIF database has been extensively used for research purposes and is described in detail elsewhere [44]. It includes the four major health insurance schemes (for salaried workers, farmers, professional soldiers and self-employed workers) and covers 98 % of the French population. Women with distant metastasis at diagnosis, cancer relapse, serious cognitive troubles, deafness, or acute psychiatric disease, and those unable to answer a questionnaire were not included. The study methodology was designed and performed in accordance with the Declaration of Helsinki, and was approved by two French national ethics commissions: the CCTIRS (Comité Consultatif sur le Traitement de l'Information en Matière de Recherche dans le Domaine de la Santé, study registered under n°05.254) and the CNIL (French Commission on Individual Data Protection and Public Liberties, study registered under n°05-1319).

### Data collection

An additional file shows the questions used in the analysis, extracted from the ELIPPSE40 cohort questionnaires [see Additional file 1].

#### Patient questionnaire

**Fig. 1:**
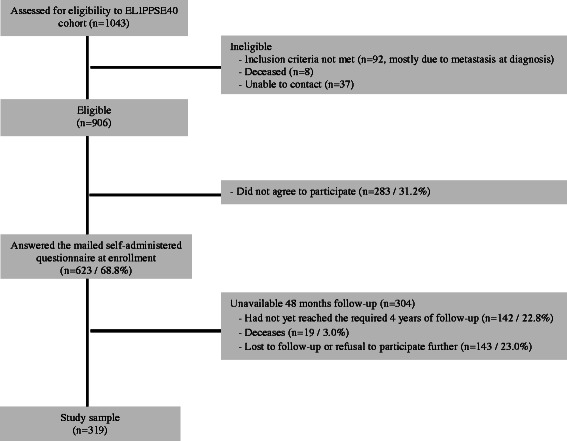
Participant flow chart

An explanatory letter about ELIPPSE40 was sent to all eligible patients (Fig. 1). Those who provided their signed written consent to participate were then mailed a first short self-administered questionnaire (directly after diagnosis, entitled M0) which included questions on sociodemographic characteristics and the circumstances of their BC diagnosis. This short questionnaire was filled in by the patients in the weeks following their diagnosis and before they initiated cancer treatment.

#### Overall perception of the cancer disclosure process

**Fig. 2:**
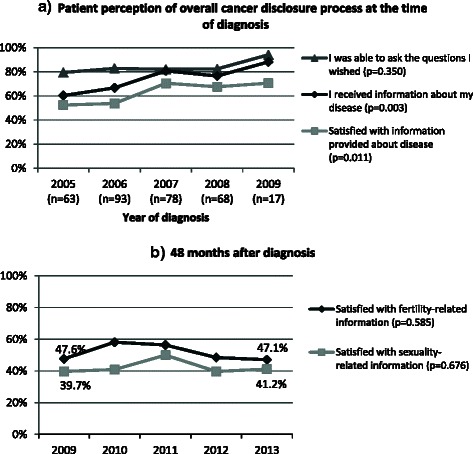
Evolution over 5 years of diagnosis of **a**) patient perception of the overall cancer disclosure process and **b**) patient satisfaction with fertility- and sexuality-related information

The first short questionnaire (at M0) included items about patient perception of the overall cancer disclosure process (Fig. 2a) coming from previous research [41, 42, 45, 46]. A first question asked patients if they were able to ask all the questions they wanted at the moment of disclosure. The answers were given on a 4-point Likert scale that was dichotomized secondarily to describe the number of participants who responded "*Yes absolutely*" or "*Yes maybe*" (versus "*No not really*" or "*No not at all*"). A second question asked about whether they had received information about their disease or not. Two other questions asked patients if the information provided was understandable and meet their expectations. These 4-point Likert scales with responses ranging from "*Yes absolutely*" to "*No not at all*" were merged and patients were considered satisfied with information provided about their disease if they responded "*Yes absolutely*" or "*Yes maybe*" to both questions.

#### Psychometric measures

Four Computer-Assisted Telephone Interviews (CATI, conducted by a trained investigator) were planned at 10, 16, 28 and 48 Months after diagnosis (entitled M10, M16, M28 and M48, respectively).

The initial mental adjustment of women after disclosure of cancer diagnosis was measured at M10 using the 21-item validated French version of the Mental Adjustment to Cancer scale (MAC-21, [47]). The MAC-21 consists of three subscales: “fighting spirit” measures positive adjustment, while “helplessness/hopelessness” and “anxious preoccupation” measure negative adjustment [47]. Anxious preoccupation reflects significant anxiety associated with diagnosis and prognosis, the latter being perceived as uncertain.

Other validated psychometric scales were also used to measure quality of life (WHOQOL-BREF, [48]) and depressive symptoms (CES-D, [49]) at all four follow-up interviews, body image (BIS, [50]) at the last three interviews, and sexual dysfunction (Relationship and Sexuality Scale (RSS), [16]) at the final interview (M48) only.

#### Satisfaction with fertility- and sexuality-related information (FRI/SRI)

Satisfaction was assessed at M48 on 5-point Likert scales with responses ranging from "strongly disagree" to "strongly agree”. The first item of the RSS (“*I think I have received sufficient information about how disease and treatment* (*including surgery*) *might affect my sexual life*”) validated by Berglund et al. [16] was chosen to measure satisfaction with Sexuality-Related Information (SRI). It was then adapted (“*I think I have received sufficient information about how treatment* (*including surgery*) *might affect my fertility*”) to measure satisfaction with Fertility-Related Information (FRI). Women who answered “*I agree*” or “*I strongly agree*” to each question were considered satisfied with FRI or SRI.

#### Medical characteristics

In parallel with patients’ telephone interviews, a first medical questionnaire (at M10) was completed by the patient’s physician who made the diagnoses and/or was in charge of cancer treatment. This questionnaire dealt with the patient’s medical history, comorbidities (from the Charlson index [51]), pathological assessment of BC (date of diagnosis, tumor size and grade, histological type, lymph nodes' status, estrogen and progesterone receptor status, and HER2⁄neu over expression), and information about treatment. We also collected financial reimbursement data for hormone therapy drugs. Overall, 99.4 % of patients had a complete medical questionnaire at M10. For the two other patients (0.6 %), missing medical information was completed with the help of subsequent medical questionnaires and financial reimbursement data.

### Statistical analysis

A Spearman’s correlation analysis was performed between FRI and SRI satisfaction, and depressive symptoms, body image and other psychosocial characteristics. Chi-squared tests (categorical characteristics) and Student's t-tests (continuous characteristics) were used to compare satisfied women with the rest of the sample. The associations between satisfaction and explanatory variables (included those just listed) were then evaluated using binary logistic regression models. All variables with a p-value (*P*) <0.20 in univariate analysis were eligible for the multivariate model. Systematic adjustment was performed for age, educational level, parenthood before diagnosis, specific consequences of cancer and its treatment (infertility or sexual dysfunction) and general satisfaction with medical follow-up at M48. Multivariate models were estimated after applying multiple imputation to the 19 (6.0 %) patients who had monotone-missing values for educational level, who were parents before diagnosis and had a history of attention and/or memory problems (Table 1). Factors associated with satisfaction were identified in the multivariate binary logistic regression model using a backward procedure (*P <*0.10). All analyses were performed using Stata software (Stata Corp., College Station, Texas, USA).

**Table 1:** Satisfaction with fertility- and sexuality-related information in 319 young breast cancer survivors

	Total	Satisfied with FRI		Satisfied with SRI	
Continuous data	Mean ± SD	Mean ± SD	*P* value	Mean ± SD	*P* value
Frequency data	n (% column)	n (% row)		n (% row)	
**At baseline (M0)**					
Age, years	36.7 ± 3.7	36.7 ± 3.7	0.658	36.7 ± 3.5	0.798
French as mother-language			0.615		0.901
No	17 (5.3)	8 (47.1)		7 (41.2)	
Yes	302 (94.7)	161 (53.3)		129 (42.7)	
Living as a couple or having regular partner			0.152		0.126
No	41 (12.9)	26 (63.4)		22 (53.7)	
Yes	278 (87.1)	143 (51.4)		114 (41.0)	
Level of education			0.977		0.086
Lower than high school graduate	103 (32.3)	54 (52.4)		53 (51.5)	
High school graduate	60 (18.8)	31 (51.7)		25 (41.7)	
Greater than high school graduate	137 (42.9)	73 (53.3)		51 (37.2)	
Missing values	19 (6.0)				
Employed			0.251		0.050
No	55 (17.2)	33 (60.0)		30 (54.5)	
Yes	264 (82.8)	136 (51.5)		106 (40.2)	
Having children			0.446		0.911
None	50 (15.7)	29 (58.0)		21 (42.0)	
One or more	259 (81.2)	135 (52.1)		111 (42.9)	
Missing values	10 (3.1)				
Place of residence			0.858		0.680
Rural	38 (11.9)	21 (55.3)		18 (47.4)	
<200,000 inhabitants	99 (31.0)	54 (54.5)		44 (44.4)	
≥200,000 inhabitants	182 (57.1)	94 (51.6)		74 (40.7)	
Had opportunity to ask questions at cancer disclosure			0.002		0.041
No	56 (17.6)	19 (33.9)		17 (30.4)	
Yes	263 (82.4)	150 (57.0)		119 (45.2)	
Received information about disease at cancer disclosure			0.124		0.006
No	89 (27.9)	41 (46.1)		27 (30.3)	
Yes	230 (72.1)	128 (55.7)		109 (47.4)	
Satisfied with information about disease at cancer disclosure			0.035		0.004
No	123 (38.6)	56 (45.5)		40 (32.5)	
Yes	196 (61.4)	113 (57.7)		96 (49.0)	
**At 10 month after diagnosis (M10): medical characteristics**					
Tumor stage			0.960		0.349
Stage 0	36 (11.3)	19 (52.8)		16 (44.4)	
Stage I	89 (27.9)	47 (52.8)		36 (40.4)	
Stage II	145 (45.5)	79 (54.5)		63 (43.4)	
Stage III	44 (13.8)	22 (50.0)		21 (47.7)	
Unknown	5 (1.6)	2 (40.0)		0 (0.0)	
Breast surgery			0.216		0.250
Mastectomy	121 (37.9)	59 (48.8)		57 (47.1)	
Tumorectomy	195 (61.1)	109 (55.9)		71 (40.5)	
Missing values	3 (0.9)				
Chemotherapy			0.769		0.752
No	70 (21.9)	36 (51.4)		31 (44.3)	
Yes	249 (78.1)	133 (53.4)		105 (42.2)	
FEC100 chemotherapy regimen			0.126		0.430
No	142 (44.5)	82 (57.7)		64 (45.1)	
Yes	177 (55.5)	87 (49.2)		72 (40.7)	
Radiotherapy			0.696		0.206
No	32 (10.0)	18 (56.3)		17 (53.1)	
Yes	287 (90.0)	151 (52.6)		119 (41.5)	
Trastuzumab treatment			0.728		0.185
No	259 (81.2)	136 (52.5)		115 (44.4)	
Yes	60 (18.8)	33 (55.0)		21 (35.0)	
Hormone treatment			0.197		0.094
No	120 (37.6)	58 (48.3)		44 (36.7)	
Yes	199 (62.4)	111 (55.8)		92 (46.2)	
Family history of breast or ovarian cancer			0.076		0.468
No	225 (70.5)	112 (49.8)		93 (41.3)	
Yes	94 (29.5)	57 (60.6)		43 (45.7)	
Report of “serious” comorbidities^a^ by physician			0.401		0.441
No	273 (85.6)	142 (52.0)		114 (41.8)	
Yes	46 (14.4)	27 (58.7)		22 (47.8)	
**At 10 month after diagnosis (M10): patient questionnaire**					
Attention and⁄or memory problems			0.232		0.037
No	99 (31.0)	57 (57.6)		51 (51.5)	
Yes	201 (63.0)	101 (50.2)		78 (38.8)	
Missing values	19 (6.0)				
MAC-21 score					
Anxious preoccupation subscale	50.0 ± 10.0	48.3 ± 10.3	0.001	48.8 ± 10.4	0.084
Fighting spirit subscale	50.6 ± 10.0	51.9 ± 9.1	0.014	51.4 ± 9.0	0.196
Hopelessness/helplessness subscale	49.2 ± 9.6	48.3 ± 8.6	0.075	49.1 ± 8.6	0.784
**At 48 month after diagnosis (M48)**					
Preserved fertility			0.478		0.429
No	221 (69.3)	120 (54.3)		91 (41.2)	
Yes	98 (30.7)	49 (50.0)		45 (45.9)	
Satisfied with medical follow-up			0.005		0.007
No	91 (28.5)	37 (40.7)		28 (30.8)	
Yes	228 (71.5)	132 (57.9)		108 (47.4)	
Score of sexual dysfunction (RSS subscale)	9.5 ± 4.1	9.2 ± 4.2	0.113	9.1 ± 4.2	0.110

*FRI* fertility-related information; *SRI* sexuality-related information; *SD* standard deviation; *MAC*-*21* 21-item mental adjustment scale to cancer; *RSS* relationship and sexuality scale

^a^Including asthma, diabetes, cardiac problems, HIV infection and/or Hodgkin’s disease

## Results

### Description of the study population

From 2005 to 2011, of the 1043 women with BC under 40 identified in the NHIF database, 906 were eligible to participate in the ELIPPSE40 cohort (Fig. 1). Among the latter, 283 (31.2 %) did not wish to participate and so 623 were included. No differences were observed in NHIF characteristics between those included and those who refused to participate in terms of age (*P* = 0.692) size (*P* = 0.509) and type (*P* = 0.501) of municipality, and administrative area (department) of residence (*P* = 0.181). As of 27 January 2014, 22.8 % had not yet completed the required 4 years (M48) of follow-up (therefore 2009 was the last full year which could be analyzed), 3.0 % were deceased and 23.0 % were lost to follow-up (or had interrupted their follow-up). Thus, analysis of FRI and SRI satisfaction was conducted among the remaining 319 women (Fig. 1). No differences were observed between these 319 women and those lost to follow-up (*n* = 143) in terms of age (*P* = 0.947), sociodemographic characteristics and tumor stage (*P* = 0.193). Overall, the comparison between these 319 women and all those without any 48 months follow-up (*n* = 304) in term of age, sociodemographic characteristics and tumor stage did not reach statistical significance. Patient characteristics for the 319 women still being followed 4 years after diagnosis are described in Table 1. Patients were followed by cancer specialist (93.7 %), Obstetrician Gynecologists (61.1 %), Psychologist/Psychiatrist (59.2 %), and/or primary care practitioners (43.9 %) over the 4 years. These consultations with different medical specialties were not statistically associated with FRI and SRI satisfaction.

### Patient perceived provision of information

Figure 2a shows the evolution of patient perception of cancer-related information provided at disclosure over the 2005–2009 period. The levels of information provided appeared to increase with each year of diagnosis (*P* = 0.003) rising from 60.3 % in 2005 to 88.2 % in 2009. Satisfaction with information provided at diagnosis also increased (*P* = 0.011): 52.4 % of women in 2005 reported that information received met their expectations. This value rose to 70.6 % in 2009. Patients’ perception about the ease of asking doctors all the questions they wished to ask was already high in 2005 and did not significantly increase over time (*P* = 0.350).

Four years after diagnosis, only 53.0 and 42.6 % of women declared that they were satisfied with information received about fertility and sexuality after cancer, respectively. A moderate correlation (Spearman’s rho (*ρ*) = 0.60; *P* <0.001) was found between these two types of information. Their correlates were different: only FRI satisfaction was associated with women’s overall perception of their health (*ρ =* 0.17; *P* = 0.003) and depressive symptoms (*ρ* = −0.15; *P* = 0.007). Deterioration of body image was significantly associated with both FRI satisfaction (*ρ* = −0.16; *P* = 0.004) and SRI satisfaction (*ρ* = −0.12; *P* = 0.031). Finally (Fig. 2b), no significant association was observed between year of diagnosis and FRI satisfaction (*P* = 0.585) or SRI satisfaction (*P* = 0.676).

### Factors associated with satisfaction with fertility-related information (FRI)

In multivariate analysis (Table 2), factors associated with lower probability of FRI satisfaction included anxious preoccupation (MAC-21 subscale). Women who reported that they had had the opportunity to ask all the questions they wished at cancer disclosure, those who reported they were satisfied in general with their medical follow-up, and finally those with a family history of breast or ovarian cancer, were all more likely to be satisfied with FRI than other women.

**Table 2:** Predictors of satisfaction with information about fertility and sexuality in 319 young breast cancer survivors

	FRI satisfied		SRI satisfied	
	OR [95 % CI]	*P* value	OR [95 % CI]	*P* value
Age at diagnosis, years	1.01 [0.95, 1.08]	0.738	1.01 [0.95, 1.08]	0.713
Living as a couple or having regular partner at diagnosis	0.50 [0.23, 1.10]	0.084	0.48 [0.23, 1.02]	0.057
Education level (versus “less than high school graduate”)				
High school graduate	0.94 [0.47, 1.86]	0.849	0.67 [0.35, 1.31]	0.245
Greater than high school graduate	0.88 [0.51, 1.54]	0.664	0.53 [0.31, 0.92]	0.023
Having children at the time of diagnosis	1.00 [0.48, 2.09]	0.996	1.18 [0.58, 2.43]	0.645
Had opportunity to ask questions at cancer disclosure	2.43 [1.27, 4.65]	0.007	NE	
Lack of information provided about disease at cancer disclosure	NE		0.54 [0.31, 0.93]	0.028
History of attention and/or memory problems at M10	NE		0.58 [0.34, 0.99]	0.045
FEC100 chemotherapy regimen	0.63 [0.39, 1.03]	0.064	NE	
Hormone treatment	1.48 [0.88, 2.51]	0.140	1.63 [0.99, 2.70]	0.056
Family history of breast or ovarian cancer	1.72 [1.01, 2.94]	0.046	NE	
Score of anxious preoccupation (MAC-21 subscale) at M10	0.97 [0.94, 0.99]	0.006	NE	
Fertility preserved at M48	0.80 [0.46, 1.40]	0.440	NE	
Score of sexual dysfunction (RSS subscale) at M48	NE		0.96 [0.90, 1.01]	0.138
Satisfied with medical follow-up at M48	2.23 [1.31, 3.79]	0.003	2.03 [1.18, 3.52]	0.011

*FRI* fertility-related information; *SRI* sexuality-related information; *OR* odds ratio (*estimated by multivariate binary logistic regression models after applying multiple imputations*); *CI* confidence interval; *NE* not entered in the regression model; *M10* 10 months after diagnosis; *M48* 48 months after diagnosis; *MAC*-*21* 21-item mental adjustment scale to cancer; *RSS* relationship and sexuality scale

### Factors associated with satisfaction with sexuality-related information (SRI)

Multivariate analysis showed that a higher level of education, a history of attention and/or memory problems and a lack of general BC information being provided at cancer disclosure were all independently and negatively associated with SRI satisfaction. By contrast, women who reported that they were very satisfied in general with their medical follow-up were more likely to be satisfied with SRI than others.

## Discussion

In this population of young BC survivors, only 53.0 and 42.6 % reported being satisfied with information provided about fertility and sexuality, respectively, 4 years after diagnosis. Despite a trend towards a general increase in and satisfaction with the information provided at cancer disclosure, these two long-term issues, particularly sexuality, remain insufficiently addressed. To our knowledge, this is the first prospective cohort study to simultaneously evaluate patient perception of information provided at cancer disclosure and the subsequent patient satisfaction with information about sexuality and fertility issues. It highlights that both these issues are different: they are moderately correlated but have distinct correlates and contributing factors. Indeed, general satisfaction with medical follow-up was the only common factor observed (Table 2). As one might expect this factor to be strongly associated with more specific aspects of satisfaction, it was systematically adjusted for in all analyses of more specific satisfaction variables.

In this study, the percentage of women who reported having had satisfactory discussions on fertility issues with their healthcare providers is quite similar to the 51.0 % of patients who felt their fertility concerns were addressed adequately in Partridge et al. [52] in the USA. However, the rate of satisfaction with information provided about effects of cancer treatments on sexuality in our study is below the 52.4 % reported by Ussher et al. [30] in their study on sexual well-being in Australian BC survivors of all ages surveyed on average 3.9 years after diagnosis. One reason may be the lack of specific guidelines concerning discussion about sexuality for cancer patients at the time of the study in France. Our result confirms the important need to provide detailed and comprehensive information on sexuality.

Differences regarding predictors associated with FRI and SRI satisfaction were also observed, particularly concerning patient proactivity when obtaining information about these two distinct issues. For example, a history of breast or ovarian cancer among family members of patients and patients’ active involvement in asking questions at cancer disclosure were beneficial to obtaining information on fertility issues. Several physician barriers to initiating discussions about fertility have been previously described [53, 54] and may explain why patients are more active and informed by family to obtain satisfactory information. Indeed, physician discussion with their patients (and families) may well be hindered by a lack of knowledge, resources and communication skills (lack of adequate vocabulary), or system barriers (the optimal time to raise these issues [24] may interfere or compete with several other issues related to the diagnosis, treatment, and prognosis that must be discussed in the already lengthy initial oncology visit). Instead SRI satisfaction was associated more with the amount of information provided spontaneously (i.e. not in response to a patient question) by the physicians at cancer disclosure, suggesting a more passive involvement of patients regarding sexuality.

Young BC survivors do not obtain adequate FRI and SRI because the healthcare providers involved tend to underestimate the importance of this information [7, 30]. In Ussher et al. [30] one patient reported: “*It took 4 or 5 times of broaching the subject with my doctors to finally get a referral to someone who could give me some advice on how to deal with the vaginal dryness*”. Thewes et al. [29] observed that physicians were slow to realize that fertility-related information really is an important issue. Indeed, it has been shown that doctors are not particularly good at eliciting symptoms related to treatment morbidity [55]. Clinicians need to be more aware of symptoms related to treatment morbidity and need to be more receptive to their patients’ need for information.

As shown in previous research, patients who feel they are poorly informed are not only dissatisfied with their care [7, 29, 30, 56], but may also experience a deterioration in psychosocial wellbeing [7, 23, 27, 28, 31, 34, 35]. The present study showed that better score of women’s perception on their body image was associated with greater satisfaction with both FRI and SRI, although a simple correlation between two variables measured at the same time cannot prove the existence of a causal relationship. Satisfaction with FRI was also negatively associated with depressive symptoms. Previous studies [31, 34, 35] showed the significant association between satisfaction with BC information and patients’ health-related physical and mental quality of life. In their study including patients and partners across a range of sexual and non-sexual cancers, Perz et al. [23] also showed that dyadic sexual communication was a significant predictor of sexual functioning among women of all ages. Thus, being provided with such information can alleviate the anxiety around post-cancer intimate changes, minimize the negative impact on intimate relationships and enhance health-related quality of life [56]. Furthermore, in the present study, only the satisfaction with FRI was correlated with women’s overall perception of their health. For young women with BC, infertility may perhaps be discussed more with healthcare providers but may also be more important for patients compared with sexual dysfunction.

In our study, no socio-demographic characteristic, including age and previous children, was associated with FRI satisfaction, although infertility concerns may be higher among young women with fewer children [52]. However, for sexuality, young women with a level of education higher than high school were less likely to be satisfied with SRI than those with a lower education level. Furthermore, women who had a sexual partner were, unsurprisingly, more upset about intimate issues, since single women who are not sexually active may not be as aware of the problems associated with intimacy and the disease [25]. This factor did not reach statistical significance.

Contrary to previous results [45, 57], the association between hormone treatment and FRI satisfaction did not reach statistical significance. Moreover, our multivariate analysis showed a non-significant difference between women who had received FEC100 chemotherapy regimen and the others, in terms of FRI satisfaction, despite the known side effects of this regimen on ovarian function [10].

Some of the factors reducing the probability of being satisfied with information provided among these women may well be beyond the scope of medical treatment, to the extent that they are related to psychosocial vulnerability [5]. Indeed, our analyses highlight that feelings of anxiety were associated with poor FRI satisfaction. Anxious preoccupation in a young woman coping with BC may stem from not knowing if she will still be able to have a child. Four years after diagnosis, most of the women in our study were over 40 years of age, and had received aggressive BC treatment, increasing the risk of fertility loss due to premature ovarian aging [10]. Having a history of attention and/or memory problems was also associated with poor SRI satisfaction, showing the inability of woman with cognitive impairment to retain information about sexual health, particularly information passively obtained (i.e. information provided spontaneously by the doctor, not as a result of patient questions) [40].

Some limitations of our study have to be acknowledged. No open-ended questions about FRI/SRI were included on the one hand, and health literacy has not been assessed, on the other. Hence issues regarding the kinds, nature, quality, content and impact of the information (that patients have accessed or understood) have not been addressed [56]. Participants in this study might also not recall all the information previously given (at diagnosis for example). Thus, their responses may have been influenced by recall bias. Furthermore, satisfaction with FRI and SRI was assessed at a single time point (M48). Only patients with irregular or absent menstrual periods after BC had been interrogated at M16 about provision of FRI before the start of treatment. Among the 179 women affected, those who reported they had received this information at M16 were more likely (*P* = 0.002) to be satisfied with FRI at M48 (60.1 %) than the others (40.3 %). This may offer some additional validity to the M48 assessment of satisfaction with FRI. A further limitation was that as data about satisfaction was collected through telephone interviews, the possibility of social desirability bias cannot be excluded. Moreover no FRI or SRI satisfaction measure was available for patients lost to follow-up 4 years after diagnosis. However, the effect of such limitations might have been to overestimate rather than underestimate satisfaction, if we hypothesize that social desirability might increase reported satisfaction with care and dropouts might be more severe patients with higher risk of side effects and dissatisfaction. The rather low rate of satisfied women in our study is consistent with previous findings. The main strengths of the study are its prospective design, a satisfactory response rate, and a regionally representative sample of young French women with BC. Because of regional recruitment, it was not possible to document what kinds of information were routinely provided to these women, but it seems that the general increase in the provision of BC information over the 2005–2009 study period was still not enough to meet the information needs of young BC survivors regarding the issues of fertility and sexuality.

## Conclusions

Despite a year-on-year general increase in information provided at cancer disclosure, nearly half of the young French 4-year breast cancer survivors in this study were not satisfied with the information provided about fertility. More alarmingly, a majority of these women had wished for more information on sexuality than they in fact received. More active involvement of patients, in the form of asking questions, was found to be beneficial to obtaining more information on fertility but not on sexuality. Encouraging patients to ask questions more often, through the use of question prompt lists for example [58], and providing more systematic standardized information could improve communication between caregivers and young breast cancer survivors, and address specific patient needs regarding fertility- and sexuality-related information. The training of health professionals on sexual issues and intimacy is also required to adequately and comprehensively advise couples.

## Additional file

Additional file 1: **Questionnaire.** Translation of the questions used in the analysis, extracted from the ELIPPSE40 cohort questionnaires. (PDF 391 kb)
