# Supplementary material for: Satisfaction with fertility- and sexuality-related information in young women with breast cancer—ELIPPSE40 cohort
Source: BMC Cancer. 2015 Aug 5;15:572. doi: 10.1186/s12885-015-1542-0 (PMC4523948; doi:10.1186/s12885-015-1542-0)
Supplement: Additional file 1: — Questionnaire. Translation of the questions used in the analysis, extracted from the ELIPPSE40 cohort questionnaires. (PDF 391 kb) [file 12885_2015_1542_MOESM1_ESM.pdf]

Translation of the questions used in the analysis, extracted from the ELIPPSE40 cohort questionnaires

Article title:

Satisfaction with fertility- and sexuality-related information in young women with breast cancer - ELIPPSE40 cohort

Journal name:

BMC Cancer

Author names:

Ali BEN CHARIF, Anne-Déborah BOUHNIK, Dominique REY, Magali PROVANSAL, Blandine COURBIERE, Bruno SPIRE and Julien MANCINI

Affiliation and e-mail address of the corresponding author:

Julien MANCINI, Aix-Marseille University, UMR912, SESSTIM, "*Cancers, Biomedicine & Society*" team, julien.mancini@univ-amu.fr

Etude Longitudinale sur l'Impact Psychosocial des Pathologies du SEin

Inserm
Institut national de la santé et de la recherche médicale

ORS
OBSERVATOIRE REGIONAL DE LA SANTE
PROVENCE-ALPES-CÔTE D'AZUR

« LONGITUDINAL STUDY OF THE PSYCHOSOCIAL IMPACT OF BREAST PATHOLOGY (PACA AND CORSICA REGIONS) »

PATIENT QUESTIONNAIRE: TELEPHONE INTERVIEWS

PERIOD: BASELINE (M0)

Socio-demographic characteristics

Year of birth: 19 /__/__/

Is French your mother-language? ❑Yes ❑ No

Are you currently living with a partner? ❑Yes ❑ No

If "No":

Do you have emotional and sexual relationship with a regular partner? ❑Yes ❑ No

How many children have you had (including adopted children)? |__|__| children, among which |__|__| adopted children

What is your highest education level?

1. You have not been to school
2. No diploma, but schooled until primary school or college
3. No diploma, but schooled beyond college
4. CEP (primary school certificate)
5. BEPC (General Certificate of Secondary Education), elementary certificate, general education certificate
6. CAP (Vocational Training Certificate), companion certificate
7. BEP (Professional Training Certificate)
8. General Baccalaureate, Advanced Certificate
9. Technological or professional baccalaureate, professional or technician's certificate, Certificate of Agricultural Education (BEA), of Commercial Education (BEC), of Industrial Education (BEI), of Hotelier Instruction (BEH), basic legal qualification
10. An undergraduate university degree, BTS (Advanced Technician's Diploma), DUT (Technical Diploma from a University Technology Institute), Diplomas related to healthcare and social services
11. Advanced University Degrees from a highly competitive public or private college or engineering college.

Do you have a job? ❑Yes ❑ No

Circumstances of cancer diagnosis

On what date was your disease diagnosed (month/year)? Month: /__/__/ Year: 20/__/__/

Who announced you the diagnosis of your disease?

❑ A generalist
❑ A gynaecologist
❑ A surgeon
❑ A radiologist
❑ A chemotherapist
❑ A radiotherapist
❑ A laboratory staff member
❑ A nurse
❑ Another person. Specify: ______________________________

At diagnosis disclosure, did you have the opportunity to ask all the questions you wished?

❑ Yes, absolutely
❑ Yes, maybe
❑ No, not really
❑ No, not at all

At diagnosis disclosure, did you receive information about your disease? ❑Yes ❑ No

If "Yes":

Would you say the information you received was understandable?

❑ Yes, absolutely
❑ Yes, maybe
❑ No, not really
❑ No, not at all

Would you say the information you received met your expectations?

❑ Yes, absolutely
❑ Yes, maybe
❑ No, not really
❑ No, not at all

Etude Longitudinale sur l'Impact Psychosocial des Pathologies du SEin

« LONGITUDINAL STUDY OF THE PSYCHOSOCIAL IMPACT OF BREAST PATHOLOGY (PACA AND CORSICA REGIONS) »

MEDICAL QUESTIONNAIRE: POSTAL SURVEY

PERIOD: DIAGNOSIS + 10 MONTHS (M10)

Tumour characteristics

Date of first suspect examination of malignancy (mammography, ultrasound…)? l__l__l / l__l__l / 20 l__l__l

Date of cancer diagnosis (month/year)? l__l__l / l__l__l / 20 l__l__l

Histological type

- ☐ Invasive ductal carcinoma
- ☐ Invasive lobular carcinoma
- ☐ Ductal carcinoma *in situ*
- ☐ Lobular carcinoma *in situ*
- ☐ Another. Specify: ____
- ☐ Unknown

pTNM clinical classification

pT – Primary Tumor

- ☐ pT0 (no evidence of primary tumor)
- ☐ pTis (carcinoma *in situ*)
- ☐ pT1 (tumor 2 cm or less in greatest dimension)
- ☐ pT2 (tumor >2 cm and $\leq$5 cm in greatest dimension)
- ☐ pT3 (tumor >5 cm in greatest dimension)
- ☐ pT4 (tumor of any size with direct extension to chest wall or skin)
- ☐ pTX (primary tumor cannot be assessed)

pN – Regional Lymph Nodes

- ☐ pN0 (no regional lymph nodes metastasis)
- ☐ pN1 (metastasis to movable ipsilateral axillary node(s))
- ☐ pN2 (metastasis to ipsilateral axillary node(s) fixed to one another or to other structures)
- ☐ pN3 (metastasis to ipsilateral internal mammary lymph node(s))
- ☐ pNX (regional lymph nodes cannot be assessed)

M – Distance Metastasis

- ☐ M0 (no distant metastasis)
- ☐ M1 (presence of distant metastasis)
- ☐ MX (metastasis cannot be assessed)

ErbB2 status: ❑ Positive ❑ Negative ❑ Unknown

Grade of Scarff-Bloom-Richardson: ❑ SBR I ❑ SBR II ❑ SBR III ❑ Unknown

Estrogen receptor: Results ____% ❑ Positive ❑ Negative ❑ No dosage ❑ Unknown

Progesterone receptor: Results ____% ❑ Positive ❑ Negative ❑ No dosage ❑ Unknown

Information about treatment

Surgery: ❑ Total mastectomy ❑ Partial mastectomy ❑ Prophylactic mastectomy ❑ Tumorectomy ❑ No

Chemotherapy: ❑ Neoadjuvant ❑ Adjuvant ❑ No

If "Yes"
Type of chemotherapy: ________________________
Number of planned cycles: l__l__l
Number of performed cycles: l__l__l

Radiotherapy: ❑ Neoadjuvant ❑ Adjuvant ❑ No

Trastuzumab: ❑Yes ❑ No

Hormone therapy: ❑Yes ❑ No

If "Yes"
Type of hormone therapy: __________________________
Posology: l__l__l mg / day Total timeline: l__l__l years

Family history of breast cancer: ❑Yes ❑ No ❑ Unknown

Family history of other cancers: ❑ No ❑ Yes. Specify if known: ________________________________

Co-morbidities

Myocardial infarction: ❑Yes ❑ No

Congestive heart failure: ❑Yes ❑ No

Peripheral vascular disease: ❑Yes ❑ No

Cerebrovascular accident (except Hemiplegia): ❑Yes ❑ No

Hemiplegia: ❑Yes ❑ No

Dementia: ❑Yes ❑ No

Chronic pulmonary disease: ❑Yes ❑ No

Rheumatic or connective Tissue Disease: ❑Yes ❑ No

Oeso-gastro-duodenal ulcers: ❑Yes ❑ No

Diabetes: ❑ Uncomplicated ❑ With documented target organ damage ❑ No

Liver hepatic: ❑ Mild ❑ Moderate or severe ❑ No

Kidney disease: ❑ Mild ❑ Moderate or severe ❑ No

Leukaemia: ❑Yes ❑ No

Lymphoma, multiple myeloma: ❑Yes ❑ No

AIDS: ❑Yes ❑ No

Others ____

History of breast pathology or surgery: ❑Unknown ❑ No ❑ Yes. Specify if known____________________

Elippse

Etude Longitudinale sur l'Impact Psychosocial des Pathologies du SEin

Inserm

Institut national de la santé et de la recherche médicale

ORS

OBSERVATOIRE REGIONAL DE LA SANTE PROVENCE-ALPES-CÔTE D'AZUR

canceropôle

Provence-Alpes-Côte d'Azur

« LONGITUDINAL STUDY OF THE PSYCHOSOCIAL IMPACT OF BREAST PATHOLOGY (PACA AND CORSICA REGIONS) »

PATIENT QUESTIONNAIRE: TELEPHONE INTERVIEWS

PERIOD: DIAGNOSIS + 10 MONTHS (M10)

Attention and/or memory problems

RESPONSES FOR EACH ITEM: 1. Very often 2. Often 3. Rarely 4. Never

Do you have attention difficulties, for example, to read or watch TV?

Do you have difficulties to memorize or remember things?

The 21-item Mental Adjustment to Cancer

INSTRUCTION FOR QUESTIONS*: the following statements describe people's reactions to having breast disease. Please indicate us, for each statement, how far it applies to you at present.*

RESPONSES FOR EACH ITEM: 1. Definitely does not apply to me 2. Does not apply to me 3. Applies to me 4. Definitely applies to me

(Anxious preoccupation)

I feel that problems with my health prevent me from planning ahead

I don't dwell on my illness

I worry about my breast disease returning or getting worse

I suffer great anxiety about it

I feel very angry about what has happened to me

(Helplessness – Hopelessness)

I feel I can't do anything to cheer myself up

I feel that nothing I can do will make any difference

I feel that life is hopeless

I feel there is nothing I can do to help myself

I am not very hopeful about the future

I feel like giving up

I feel completely at a loss about what to do

(Fighting spirit)

I believe that my positive attitude will benefit my health

I firmly believe that I will get better

I have plans for the future, e.g. holiday, jobs, housing

I think my state of mind can make a lot of difference to my health

I try to carry on my life as I've always done

I try to keep a sense of humour about it

I try to have a very positive attitude

I count my blessings

I try to fight the illness

Etude Longitudinale sur l'Impact Psychosocial des Pathologies du SEin

Institut national de la santé et de la recherche médicale

OBSERVATOIRE REGIONAL DE LA SANTE PROVENCE-ALPES-CÔTE D'AZUR

« LONGITUDINAL STUDY OF THE PSYCHOSOCIAL IMPACT OF BREAST PATHOLOGY (PACA AND CORSICA REGIONS) »

PATIENT QUESTIONNAIRE: TELEPHONE INTERVIEWS

PERIOD: DIAGNOSIS + 16 MONTHS (M16)

Fertility-related information

Currently, your menstruations are: 1. Regular 2. Irregular 3. Absent menstrual periods

If "Irregular or absent menstrual periods"
This phenomenon occurred: 1. Before my breast disease 2. Since my breast disease and its treatments

If "Since my breast disease and its treatments"
Before treatment, have you been clearly informed about how it might affect your fertility? 1. Yes 2. No

cancéropôle
Provence-Alpes-Côte d'Azur

Elippse

Etude Longitudinale sur l'Impact Psychosocial des Pathologies du SEin

Inserm
Institut national de la santé et de la recherche médicale

ORS
OBSERVATOIRE REGIONAL DE LA SANTE
PROVENCE-ALPES-CÔTE D'AZUR

« LONGITUDINAL STUDY OF THE PSYCHOSOCIAL IMPACT OF BREAST PATHOLOGY (PACA AND CORSICA REGIONS) »

PATIENT QUESTIONNAIRE: TELEPHONE INTERVIEWS

PERIOD: DIAGNOSIS + 48 MONTHS (M48)

Satisfaction with fertility- and sexuality-related information

RESPONSES FOR EACH ITEM: 1. Strongly disagree 2. Disagree 3. Neither agree nor disagree 4. Agree 5. Strongly agree

I think I have received sufficient information about how disease and treatment (including surgery) might affect my sexual life

I think I have received sufficient information about how treatment (including surgery) might affect my fertility

Sexual dysfunction

My sexual life has been negatively affected after the initiation of my breast disease

1. Not at all 2. Slightly 3. Rather much 4. Much 5. Very much 6. Inadequate question

My breast disease has affected my sexual desire (libido)

1. Increased 2. No change 3. Decreased 4. All gone 5. Inadequate question

My treatment has affected my sexual desire (libido)

1. Increased 2. No change 3. Decreased 4. All gone 5. Inadequate question

The frequency of sexual intercourse has changed now as compared to before my breast disease

1. Increased a lot 2. Somewhat increased 3. No change 4. Somewhat decreased 5. Decreased a lot 6. Inadequate question

My possibility to reach orgasm has changed now as compared to before my breast disease

1. Increased a lot 2. Somewhat increased 3. No change 4. Somewhat decreased 5. Decreased a lot 6. Inadequate question

Quality of life (WHOQOL-BREF)

How would you rate your quality of life?

1. Very poor 2. Poor 3. Neither poor nor good 4. Good 5. Very good

How satisfied are you with your health?

1. Very dissatisfied 2. Dissatisfied 3. Neither satisfied nor dissatisfied 4. Satisfied 5. Very satisfied

Body Image Scale

INSTRUCTION FOR QUESTIONS: *in this questionnaire you will be asked how you feel about your appearance, and about any changes that may have resulted from your disease or treatment. Please listen each item carefully, and give the reply which comes closest to the way you have been feeling about yourself, during the past week.*

RESPONSES FOR EACH ITEM: 1. Not at all 2. A little 3. Quite a bite 4. Very much

Have you been feeling self-conscious about your appearance?

Have you felt less physically attractive as a result of your disease or treatment?

Have you been dissatisfied with your appearance when dressed?

Have you been feeling less feminine as a result of your disease or treatment?

Did you find it difficult to look at yourself naked?

Have you been feeling less sexually attractive as a result of your disease or treatment?

Did you avoid people because of the way you felt about your appearance?

Have you been feeling the treatment has left your body less whole?

Have you felt dissatisfied with your body?

Have you been dissatisfied with the appearance of your scar? (add the response: 5. Not applicable)

Depressive symptoms (CES-D)

INSTRUCTION FOR QUESTIONS: *the following impressions are felt by most people. Please tell me often you have felt or behaved this ways during the past week.*

RESPONSES FOR EACH ITEM: 1. Never, very rarely (under 1 day) 2. Occasionally (1-2 days) 3. Quite often (3-4 days) 4. Frequently, all time (5-7 days)

I was bothered by things that usually don't bother me

I did not feel like eating, my appetite was poor

I felt that I could not shake off the blues even with help from family or friends

I felt that I was just as good as other people

I had trouble keeping my mind on what I was doing

I felt depressed

I felt that everything I did was an effort

I felt hopeful about the future

I thought my life had been a failure

I felt fearful

My sleep was restless

I was happy

I talked less than usual

I felt lonely

People were I enjoyed life unfriendly

I enjoyed life

I had crying spells

I felt sad

I felt that people dislike me

I could not get going

Other questions

Are you satisfied with the medical follow-up for your disease?

1. Yes, absolutely 2. Yes, rather 3. No, not really 4. No, not at all

Currently, regarding your fertility:

1. You cannot have children, specify (oophorectomy, hysterectomy, tubal ligation, other)
2. You cannot have children for the time being, specify (medical castration, chemotherapy, hormone therapy)
3. You can have children
4. Other situation specify (especially if medical recommendation to wait for a pregnancy)
5. Do not know
